# A Single Dose of Baicalin Has No Clinically Significant Effect on the Pharmacokinetics of Cyclosporine A in Healthy Chinese Volunteers

**DOI:** 10.3389/fphar.2019.00518

**Published:** 2019-05-14

**Authors:** Ruijuan Liu, Xia Li, Jingyao Wei, Shuaibing Liu, Yuanyuan Chang, Jiali Zhang, Ji Zhang, Xiaojian Zhang, Uwe Fuhr, Max Taubert, Xin Tian

**Affiliations:** ^1^ Department of Pharmacy, The First Affiliated Hospital of Zhengzhou University, Zhengzhou, China; ^2^ Henan Key Laboratory of Precision Clinical Pharmacy, Zhengzhou, China; ^3^ Department I of Pharmacology, Faculty of Medicine and University Hospital Cologne, Center for Pharmacology, University of Cologne, Cologne, Germany

**Keywords:** cyclosporine A, baicalin, pharmacokinetics, non-compartmental analysis, population pharmacokinetics, healthy volunteers

## Abstract

Despite its narrow therapeutic window and large interindividual variability, cyclosporine A (CsA) is the first-line therapy following organ transplantation. Metabolized mainly by CYP3A and being a substrate of P-glycoprotein (P-gp), CsA is susceptible to drug–drug interactions. Baicalin (BG) is a drug used for adjuvant therapy of hepatitis in traditional Chinese medicine. Since its aglycone baicalein (B) inhibits CYP3A and P-gP, co-administration might affect CsA pharmacokinetics. This study investigated the effect of BG on CsA pharmacokinetics. In a two-period study, 16 healthy volunteers received a single 200 mg oral CsA dose alone (reference period) or in combination with 500 mg BG (test period). Pharmacokinetic evaluation of CsA was carried out using non-compartmental analysis (NCA) and population pharmacokinetics (popPK). Treatments were compared using the standard bioequivalence method. Based on NCA, 90% CIs of AUC and *C*_max_ test-to-reference ratios were within bioequivalence boundaries. In the popPK analysis, a two-compartment model (clearance/F 62.8 L/h, central and peripheral volume of distribution/F 254 L and 388 L) with transit compartments for absorption appropriately described CsA concentrations. No clinically relevant effect of 500 mg BG co-administration on CsA pharmacokinetics was identified and both treatments were well tolerated.

## Introduction

As an immunosuppressant drug, cyclosporine A (CsA) has been widely used in transplantation since the early 1980s (Colombo and Ammirati, 2011). From then on, CsA remained a first-line therapy for patients with solid organ transplantation. However, CsA has a narrow therapeutic range and large interindividual pharmacokinetic variability. While underexposure might cause graft versus host disease (Rogosheske et al., 2014) and acute rejection episodes, overexposure might result in toxicity (Bardazzi et al., 2018). CsA is categorized as a biopharmaceutical classification system (BSC) class II drug due to low solubility and high permeability (Onoue et al., 2010). After oral administration, CsA is absorbed from the gastrointestinal tract with a bioavailability of approximately 30% (Drewe et al., 1992). As a lipophilic molecule, CsA has a high volume of distribution (3–5 L/kg); in blood, cyclosporine is extensively bound to erythrocytes. In plasma, approximately 90% is bound to proteins, primarily lipoproteins (U.S. Food and Drug Administration, 2015). The disposition of cyclosporine is generally biphasic, with a terminal half-life of approximately 8.4 h (range 5–18 h) (U.S. Food and Drug Administration, 2015). Cyclosporine is extensively metabolized by CYP3A4 and CYP3A5 and subject to efflux from renal tubular cells and other cells *via* P-glycoprotein (P-gp) (Hebert, 1997). Therefore, co-administration of CYP3A or P-gp inhibitors may alter the pharmacokinetics of CsA. For example, concomitant administration of ketoconazole has been reported to elevate CsA concentrations several-fold (Albengres and Tillement, 1992; Keogh et al., 1995). Imatinib, a potent inhibitor of CYP3A4 and P-gP, approximately doubled CsA exposure (Atiq et al., 2016).

Baicalin (baicalein 7-O-glucuronide, BG), the major bioactive compound from *Scutellaria baicalensis* (Shi et al., 2016), is widely applied in traditional Chinese medicine for the treatment of inflammation, hepatitis, various infections, and tumors (Xi et al., 2015; Zhao et al., 2016; Gong et al., 2017; Ming et al., 2018). In 2005, baicalin capsules (250 mg per capsule, approval no. H20158009) were approved by the state food and drug administration of China for the adjuvant therapy of hepatitis (2 capsules 3 times a day). After oral administration, BG is rapidly hydrolyzed to baicalein (B) by β-glucuronidase derived from intestinal bacteria (Huang et al., 2019). Both BG and its aglycone baicalein (B) have a low hydrophilicity (solubility of BG is 0.057 mg/ml in water) (Wu et al., 2011) and a relatively low permeability as determined in the Caco2 cell system [for BG, Papp = (0.275 ± 1.14) × 10^−6^ cm/s (Zhu et al., 2013); for B, Papp = 9.0 × 10^−6^ cm/s (Cai et al., 2016)], resulting in a very low oral bioavailability for both baicalin (2.2%) and baicalein (Wu et al., 2014). Compared to BG, B could be better absorbed in the gastrointestinal tract and then conjugated to BG in the gut wall and liver (Liu et al., 2009; Zhang et al., 2011). BG is extensively bound to proteins (86–92%) in human plasma (Tang et al., 2006), has a short elimination half-life (6.36 ± 5.85 h), and undergoes extensive metabolism (Noh et al., 2016).

Inflammation is a significant problem in organ transplant patients. With the anti-inflammatory and antioxidant properties of BG and its aglycone B, co-administration of BG might benefit the organ transplant patients treated with CsA (Shieh et al., 2000; Dinda et al., 2017). However, several lines of evidence suggest that BG may cause drug–drug interactions in humans. B is an inhibitor of CYP3A and P-gp in rats (Morisaki et al., 2013; Miao et al., 2016). For example, intravenous administration of high BG doses (0.23–0.90g/kg) to rats decreased the clearance of midazolam by up to 43% (Xin et al., 2013). In human liver microsomes, B was reported to potently inhibit CYP3A4 (Ki for mixed-type inhibition of bufalin 5′-hydroxylation 2.3 μM; IC_50_ for inhibition of midazolam and nifedipine at their Km concentrations 13 and 15 μM, respectively) (Li et al., 2018). On the other hand, it has been reported that B (but not BG) may activate the human pregnane X receptors and the human constitutive androstane receptor (CAR) (Morisaki et al., 2013; Cheng et al., 2014; Miao et al., 2016), which could mediate CYP3A and P-gp induction. A recent study in rats indeed showed that single intravenous dose and multiple doses of BG had no effect on the pharmacokinetics of CsA, while oral administration significantly decreased *C*_max_ and AUC_0-∞_ of CsA. This indicates that BG might affect intestinal absorption and/or secretion of CsA. Further study revealed that after multiple oral doses of BG treatment, the expression of P-gp of rats increased in the intestine, but was not changed in the liver (Tian et al., 2019). Beyond affecting CYP3A and P-gp, co-administration of BG also changed plasma protein binding and apparent volumes of distribution of nifedipine, another CYP3A probe, in rats (Cheng et al., 2014). An early study in rats which directly investigated the effects of *Scutellaria* radix decoction, BG, or B on CsA pharmacokinetics provided mixed findings: the decoction reduced exposure to oral but not to intravenous CsA, while BG and even more so B increased exposure to oral CsA (Lai et al., 2004).

These results indicate that indeed co-medication with BG might alter the pharmacokinetics of CsA in humans and also indicate that any respective DDIs may be mediated by several mechanisms. So far, no clinical studies have been reported on drug–drug interactions between BG and CsA. The aim of the current study, therefore, was to explore a potential effect of BG on CsA exposure in healthy volunteers and to assess possible effects on individual pharmacokinetic processes in detail.

## Materials and Methods

### Chemicals and Reagents

Cyclosporine soft capsules (25 mg, trade name: Sandimmun Neoral) were obtained from Novartis Pharma (Basle, Switzerland); this preparation is an immediate release microemulsion. BG capsules (250 mg, trade name: Jinmeiji) were purchased from Dongguan Jinmeiji pharmaceutical company (Dongguan, China). The reference standards of cyclosporine A and cyclosporine D were purchased from Toronto Research Chemicals (Toronto, Canada). All chemicals and solvents were of HPLC grade.

### Study Population

After approval by the Ethics Committee of the first affiliated hospital of Zhengzhou University (Henan, China; approval no. SR201509), the clinical trial was performed at this hospital in accordance with the standards of Good Clinical Practice, all applicable regulations, specific legal requirements, and ethical principles as described in the Declaration of Helsinki. Subjects provided written informed consent after a comprehensive explanation of the study protocol and before any procedure was performed.

Sixteen healthy Chinese participants (8 males and 8 females, age range 19–34 years, body mass index 19.4–25.6 kg/m^2^) were enrolled in the study. Based on an intraindividual coefficient of variation of not more than 19% for CsA AUC and *C*_max_ (Avramoff et al., 2007), this sample’s size would be appropriate to assess absence of an interaction with alpha = 0.05 and a power of 90% if the true ratios for the test over reference were in the 0.95–1.05 range. Participants were ascertained to be mentally and physically healthy by medical history, clinical examination, electrocardiography, and routine laboratory analyses consisting of hematology, blood chemistry, urine screening for illicit drugs, and a quantitative pregnancy test in women to exclude pregnancy. Main exclusion criteria included: excessive smoking (more than five cigarettes per day); alcohol intake exceeding 25 g per week; a history of clinically significant cardiovascular, renal, hepatic, pulmonary, gastrointestinal, or psychiatric diseases; a history of known allergy or intolerance to any drugs; a history of drug abuse; abnormalities in clinical laboratory parameters; donating of blood or losing blood within 3 months; and suffering from any organ damage within the previous 3 months. Subjects were required to abstain from using medications, alcohol, cigarettes, and from food and beverages containing grapefruit within 2 weeks before the first dose of study medications and during the study.

### Study Design

The study was a single center, open-labeled, two-period, fixed-sequence clinical trial. All eligible subjects were admitted to the clinical trial institution and were offered a standard dinner 1 day before the trial. After overnight fasting, the participants were administrated 200 mg CsA orally (eight soft capsules) together with 240 ml of water in the morning on day 1 during the first period. Water intake was allowed 2 h after administration of the drug. All participants were given a standardized meal 4 h after dosing. In the second period, after a washout period of 2 weeks, the same procedure was repeated with CsA in combination with 500 mg BG (two capsules).

### Blood Sampling

Blood samples (4 ml each) were collected at multiple time points pre-dose, 0.5, 1.0, 1.5, 2, 2.5, 3, 4, 6, 8, 10, 12, 16, 24, 36, and 48 h after dosing on days 1 and 15, respectively. Blood samples were withdrawn using vacuum tubes containing EDTA-K_2_ and immediately transferred to labeled tubes. The samples were stored at −80°C for subsequent analysis.

### Quantification of Cyclosporine A in Blood

The analytical method for quantification of CsA in blood samples was validated according to the pertinent U.S. FDA guideline (U.S. Food and Drug Administration, 2018). Processing of the whole blood samples involved a two-step protein precipitation. Fifty microliters of zinc sulfate (10 mM) were added to 50 μl of a sample. After vortexing, internal standard (IS) cyclosporine D and 800 μL of methanol-acetonitrile (v:v = 1:1) were added and vortexed. After centrifugation, an aliquot (2 μl) of the supernatant was then injected onto an ultra high-performance liquid chromatography coupled with tandem mass spectrometry (UHPLC–MS/MS) device for analysis. UHPLC–MS/MS was performed using an ExionLC™ analytical UHPLC system (AB Sciex, MA, USA), coupled with a Qtrap 4,500 mass spectrometer (AB Sciex, Framingham, MA, USA), equipped with the Turbo lonSpray interface. Chromatographic separation was performed on a Waters (Dublin, Ireland) BEH C_18_ 2.1 mm × 100 mm, 1.6 μm column, eluted with a mobile phase consisting of mobile phase A (water with 0.1% formic acid and 2 mM ammonium acetate) and B (methanol with 0.1% formic acid) at a flow rate of 400 μl/min. The gradient elution was 0–0.8 min 65% B; 0.81–3.9 min 100% B; 4.0–5.0 min 65% B. Retention times for CsA and IS were 2.49 and 2.54 min, respectively. The protonated analyte ions were detected in positive ionization and multiple reaction monitoring modes. The mass transition pairs of m/z 1220.0 → 1202.8 and 1234.0 → 1216.8 were used to detect CsA and IS. The declustering potentials of CsA and IS were both 60 eV; the entrance potentials were 3 and 6 eV; the collision cell exit potentials were both 30 eV; and the collision energy was 23 and 22 eV, respectively. Calibration curves were linear over the concentration range of 10–3,000 ng/ml. Intra-day and inter-day coefficients of variation were lower than 7.43% in terms of relative standard deviation for the lower limit of quantification (LLOQ) and for low, medium, and high concentration quality control samples of CsA. The mean accuracy was within ±7.0% in terms of relative error for CsA. The LLOQ was 10 ng/ml.

### Safety and Tolerability Assessments

For enrolled volunteers, safety and tolerability of CsA when given alone or in combination with BG were assessed throughout the study by monitoring adverse events (AEs), standard clinical laboratory tests (clinical biochemistry, urinalysis, hematology), physical examinations, vital signs, and 12-lead electrocardiograms (ECGs). A follow-up visit was conducted about 10 days following the last dose of study medication.

### Non-compartmental Analysis

To directly assess the quantitative effect of BG on CsA exposure, standard non-compartmental analysis by use of the WinNonlin 7.0 software (Pharsight, St Louis, MO, USA) was applied to determine pharmacokinetic parameters of CsA in the periods with and without BG co-administration. Statistical analyses were performed by use of SPSS software version 11.5 (SPSS, Inc., Chicago, IL, USA). Exploratory statistical comparisons of pharmacokinetic parameters between male and female subjects were performed by the *t*-test for independent data. A nonparametric test was used to compare *T*_max_ between male and female subjects, and between the reference and BG treatment. *p* < 0.05 was considered a significant difference. To compare exposure between treatments, point estimates and 90% confidence intervals (CIs) of the geometric mean ratios of AUC and *C*_max_ of treatment over reference were used. No relevant effect of BG on CsA exposure was assumed if 90% CIs of the geometric mean of test-to-reference ratios for these parameters were within the range of 0.80–1.25. Respective descriptive comparisons were also made for further pharmacokinetic parameters where appropriate.

### Population Pharmacokinetic Analysis

#### Basic Population Pharmacokinetic Analysis

To assess potential effects of BG co-administration on individual pharmacokinetic processes in detail, a population pharmacokinetic nonlinear mixed-effects model was developed with NONMEM 7.4.1 (Icon Development Solutions, Ellicott City, MD, USA). Data preparation and graphical data visualization were conducted using R 3.4.2 (R Foundation for Statistical Computing, Vienna, Austria). Model diagnostics were performed with XPOSE 4.5.0.9. The toolkit Perl-speaks-NONMEM (PsN) (Lindbom et al., 2004) served as an application programming interface to NONMEM to aid model development and evaluation. The structural pharmacokinetic model was built step by step, beginning with a one-compartment model with linear elimination kinetics and expanded up to a three-compartment model. Interindividual (IIV) and inter-occasion variability were tested, and additive, proportional, and combined error models were evaluated. Model selection was based on a change of 3.84 points in the objective function value (OFV) being considered as statistically significant with *p* < 0.05. The Akaike Information Criteria (AIC) were compared to select non-nested models.

#### Absorption Model Selection

The absorption process of CsA is complicated and influenced by many physiological factors. It seemed that the previously published conventional absorption models (first- or zero-order, with or without lag time) were not appropriate to optimally describe the absorption profiles in the present study. Thus, other additional absorption models were tested, including Weibull-type function models, Gaussian density function models, erlang-type absorption, and transit compartment models. The model selection was based on both visual (goodness of fit plots) and numerical (OFV and AIC) procedures.

#### Covariate Selection

Based on previous knowledge on CsA pharmacokinetics, demographic and clinical variables such as age, weight, sex, hematocrit, alanine aminotransferase (ALT), aspartate aminotransferase (AST), total bilirubin, and albumin were tested for covariates analysis on PK parameters. Visual (parameter vs. covariate scatter plots) covariate screening procedures were first performed before adding each covariate to the basic model. The Stepwise Covariate Model with both forward and backward selection was further used to analyze covariates. The criteria for integration of covariates were a decrease in OFV > 3.84 (*p* < 0.05) in the forward selection and an increase in OFV > 6.64 (*p* < 0.01) in the backward selection (approximate to *χ*^2^ distribution, $\chi_{0.05,1}^{2}$ = 3.84; $\chi_{0.01,1}^{2}$ = 6.64).

#### Effect of Baicalin on the Pharmacokinetics of Cyclosporine A

An effect of BG was introduced on the following pharmacokinetic parameters of the final model of CsA with Eq. 1: number of transit compartments (N), mean transit time (MTT), absorption rate constant (Ka), apparent clearance (CL/F), Vc/F (apparent central volume of distribution), Vp/F (apparent peripheral volume of distribution), and Q/F (apparent intercompartmental clearance). A bootstrap analysis was conducted for each model. For an effect of a covariate as a factor on an individual parameter of CsA to be considered as potentially clinically relevant, both the 95% CIs for BG effects from the 1,000 bootstrap results must not include unity (Ravva et al., 2009), and the 95% would need to be at least partially outside a 0.80–1.25 range.

(1) $$PAR=\mathrm{TVPAR}\;\times \;\theta_{PAR}^{\mathrm{test}}$$

Eq. 1 PAR population pharmacokinetic parameter, representing N, MTT, Ka, CL/F, Vc/F, Vp/F, Q/F, respectively; TVPAR population median of each parameter; test (test, 0 = CsA alone, 1 = co-administration with BG), *θ*^test^ the effect of co-administration with BG on each parameter.

### Model Evaluation

Reliability and precision of model parameter estimates were confirmed by comparison to nonparametric medians and 95% CIs obtained from bootstrap statistics with 1,000 samples generated by resampling individuals with replacement (Ette, 1997; Parke et al., 1999; Lindbom et al., 2005). The model trend and variability were confirmed by visual predictive checks (VPC) performed by simulating 1,000 replicates of the original study design (Post et al., 2008).

## Results

### Non-compartmental Analysis

**Figure 1:**
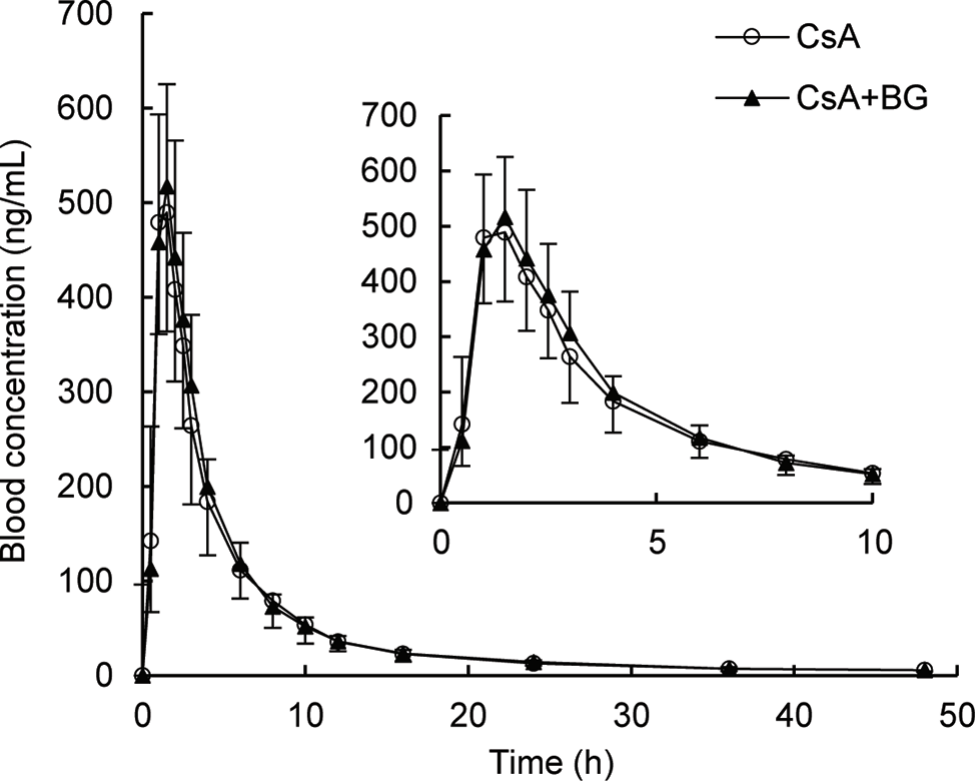
Geometric mean blood concentration-time profile of CsA in 16 healthy volunteers after single oral administration of 200 mg CsA alone or co-administration with 500 mg BG.

Mean blood concentration-time profiles of CsA are presented in Figure 1. These were nearly identical in the reference and treatment periods. Non-compartmental pharmacokinetic parameters of CsA for both periods are summarized in Table 1. Parameters were similar for test and reference periods, with point estimates for geometric mean test/reference ratios for AUC_0–48_, AUC_0-∞_, *C*_max_, t_1/2_, MRT_0–48_, CL/F, and Vz/F ranging from 0.97 to 1.03. The 90% CIs of geometric mean ratios of treatment to reference for AUC_0–48_, AUC_0-∞_, and *C*_max_ as the key parameters describing CsA exposure were within the standard bioequivalence interval of 80–125%, indicating that CsA exposure was not affected by co-administration with BG in this study. In addition, no significant differences (*p* > 0.05) were observed in *C*_max_, AUC_0–48_, AUC_0-∞_, *T*_max_, t_1/2_, MRT_0–48_, CL/F, and Vz/F of CsA between male and female subjects.

**Table 1:** Pharmacokinetic parameters of CsA after single oral administration of 200 mg CsA alone or co-administration with 500 mg BG in 16 healthy individuals (non-compartmental analysis).

Parameters	CsA alone (R)	CsA + BG (T)	T/R ratio:point estimate (90% CI)
*AUC*_0–48_ (h · μg/ml)	2.19 (19.7%)	2.22 (24.8%)	101% (88.4–116%)
*AUC*_0-∞_ (h · μg/ml)	2.33 (18.9%)	2.38 (24.4%)	102% (89.1–116%)
*C*_max_ (ng/ml)	541 (15.7%)	558 (17.5%)	103% (93.1–114%)
*T*_max_ (h)	1.5 (1.0–2.5)	1.5 (1.0–2.5)	–
*t*_1/2_ (h)	7.44 (23.7%)	7.60 (37.4%)	102% (83.0–126%)
*MRT*_0–48_ (h)	5.42 (22.5%)	5.24 (14.2%)	96.6% (86.7–108%)
*CL/F* (L/h)	85.7 (19.5%)	84.2 (26.4%)	98.2% (86.0–112%)
*Vz/F* (L)	920 (24.4%)	924 (29.7%)	100% (83.8–120%)

Data are presented as geometric mean with coefficient of variation (CV) and T_max_ as median (range). Abbreviations are as follows: CI, confidence interval; AUC_0–48_, AUC from time 0 to 48 h after administration; AUC_0-∞_, AUC extrapolated to infinity; C_max_, maximum observed blood concentration; F, (unknown) bioavailability; T_max_, time to reach C_max_; t_1/2_, apparent terminal elimination half-life; MRT_0–48_, mean residence time from 0 to 48 h; CL/F, apparent clearance; Vz/F, apparent volume of distribution during terminal phase; T/R ratio, geometric mean of parameter values CsA + BG to CsA alone.

### Population Pharmacokinetic Analysis

#### Model Development

**Figure 2:**
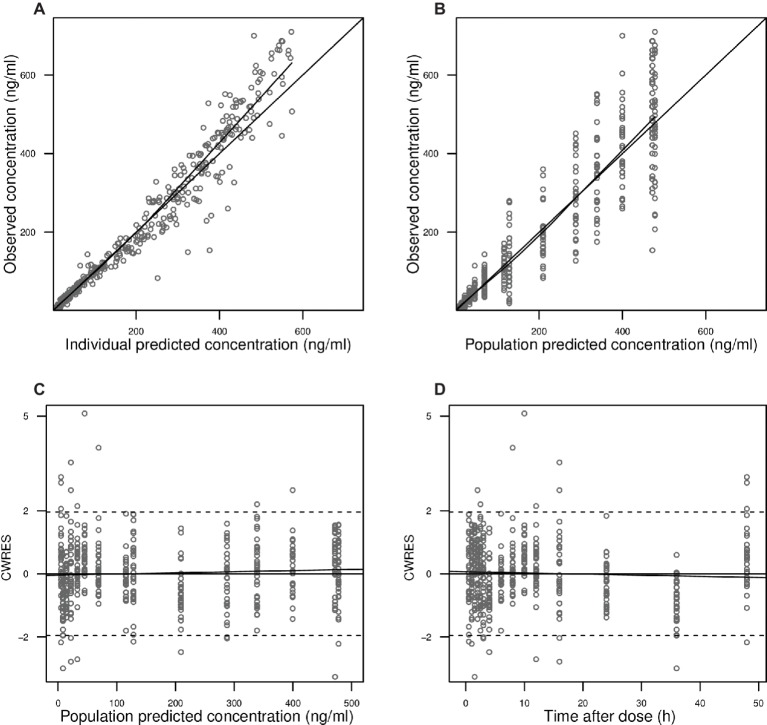
Goodness-of-fit plots. Observed blood CsA concentrations versus individual predictions **(A)** and population predictions **(B)** as obtained from the pharmacokinetic model. Conditional weighted residuals (CWRES) versus population predicted blood CsA concentrations **(C)** and versus time after the first dose **(D)**.

A two-compartment model with linear elimination with a proportional error model was selected as the structural model. Compared to other absorption models, the transit compartment model provided a statistically significant improvement in the fit (lowest OFV and AIC) and the best performance in the visual exploration of diagnostic plots (Figure 2). Although zero-order absorption with a lag time including IIV on lag time also described the absorption phase well, the bootstrap analysis indicated this absorption model was not stable, as 729 of 1,000 runs were unsuccessful. In contrast, the bootstrap analysis confirmed that the transit model with IIV or interoccasional variability (IOV) for MTT and N is stable. In comparison to an abrupt switch of the absorption rate at a certain point of time for the lag time model, the transit model more closely reflects physiological conditions with a gradually increasing absorption rate over time. Introduction of IOV for MTT and N improved the model significantly (OFV reduced by 35.03 points and 74.30 points, respectively). IIV was estimated on CL, Ka, Q, and N, leading to a significant drop in OFV by 141.0, 47.9, 35.2, and 4.4 points, respectively. Pharmacokinetic parameter estimates of the final model are presented in Table 2. Although several demographic and clinical parameters (age, weight, sex, hematocrit, ALT, AST, total bilirubin, and albumin) were tested as potential covariates on PK parameters, no significant covariate was identified with both visual and numerical covariate screening procedures (e.g., Stepwise Covariate Model).

**Table 2:** Comparison of the tested absorption models for the pharmacokinetics of CsA in the population pharmacokinetic analysis.

Model	Model tested	OFV	AIC
M1	First-order absorption	3555.31	3577.31
M2	First-order absorption with lag time	3361.25	3387.25
M3	Zero-order absorption	3446.47	3468.47
M4	Zero-order absorption with lag time	3359.28	3386.37
M5	Weibull function	3358.69	3384.69
M6	Gaussion function	3365.05	3391.04
M7	Erlang frequency (five sequential compartments)	3364.35	3388.35
M8	Transit compartment	3331.80	3357.80

#### Model Evaluation

**Figure 3:**
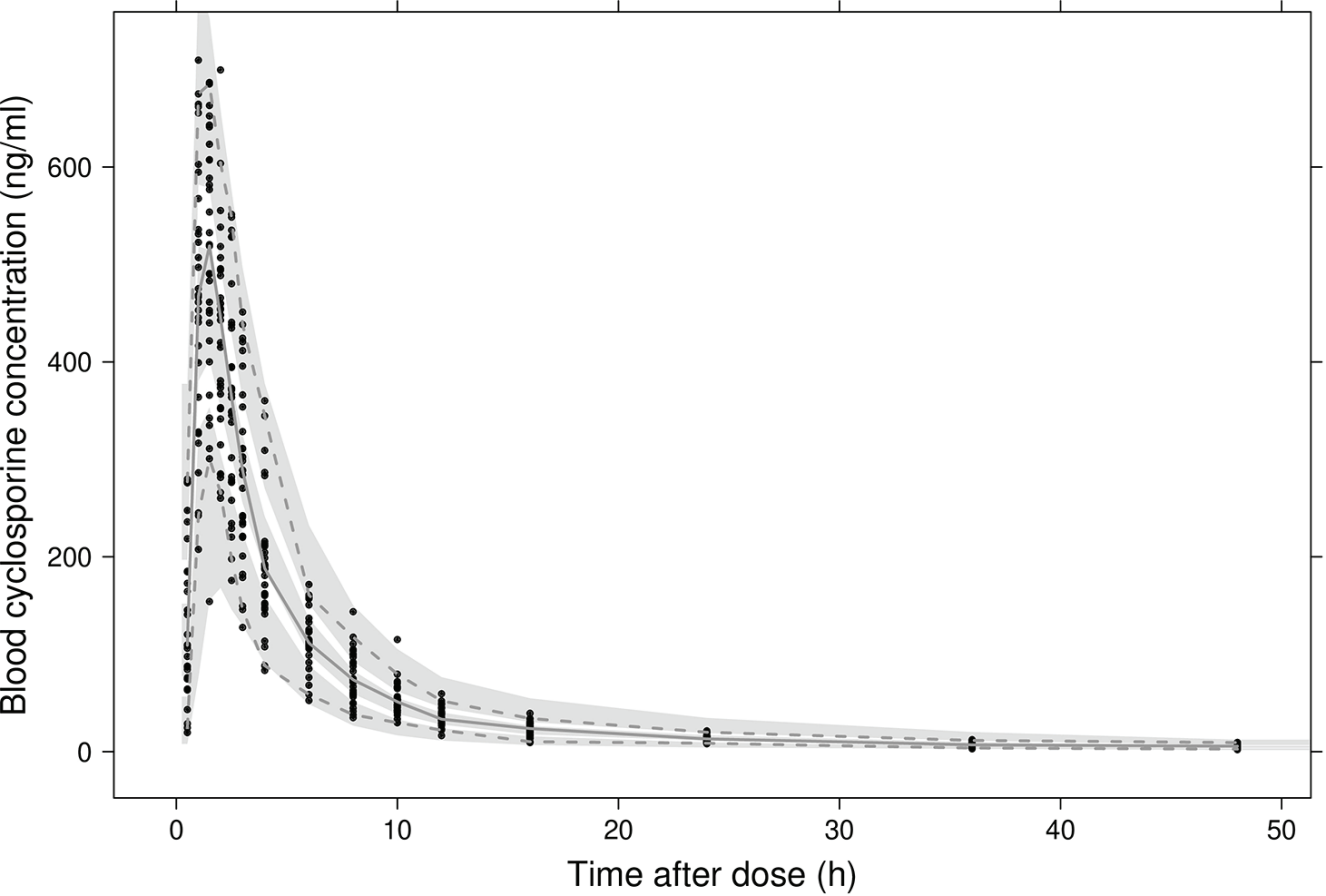
Visual predictive checks of the final model. Black dots represent observed concentrations. The solid line represents the median observed blood concentrations while 2.5th and 97.5th percentiles of the data are represented by dashed lines. Shaded areas indicate 95% intervals simulated from the model.

In Figure 2A, observed blood CsA concentrations versus individual predicted CsA concentrations exhibited a slight underestimation for high concentrations, while the weighted residual plot (Figures 2C,D) indicated that this misspecification would be acceptable because most of the residuals fell within +2 and −2 units of the null ordinate. The conditional weighted residuals (CWRES) plots (Figure 2D) appeared to show misspecification, but individual CWRES revealed that this was due to the high variability of the pharmacokinetics of CsA. While it appears that the introduction of additional compartments might attenuate the apparent misspecification, this was not supported by the respective statistical criteria. In addition, the goodness-of-fit plots of the population pharmacokinetic model for CsA in each treatment further indicated that the final model fit well the observed concentration-time profile of CsA for both treatments. In the VPC of the final population PK model with the transit compartments, medians and 2.5th and 97.5th percentiles of the simulated data were in good agreement with the observed data, verifying the good performance of this model (Figure 3). The median parameter estimates and 95% CI obtained from bootstrap are summarized in Table 3.

**Table 3:** Pharmacokinetic parameter estimates of the final population pharmacokinetic model of CsA.

Parameter (unit)	Definition of parameter	Bootstrap	
		Median	95% CI^a^
**𝜽−*Estimates***			
*CL / F* (*l / h*)	Apparent clearance	62.8	(54.4–71.2)
*Vc / F* (*l*)	Apparent central volume of distribution	254	(226–281)
*Vp / F* (*l*)	Apparent peripheral volume of distribution	388	(344–456)
*Q* (*l / h*)	Intercompartmental clearance between central and peripheral compartment	23.6	(19.3–29.5)
*Ka*(*h*^-1^)	Absorption rate constant	12.4	(6.57–33.9)
*MTT* (*h*)	Mean transit time	0.812	(0.797–0.831)
*N*	Number of transit compartment	20.2	(16.6–25.6)
**𝝎^2^−*Estimates***			
*IIV CL* (*CV%*)	Interindividual variability on clearance	12.6	(3.69–20.4)
*IIV Ka* (*CV%*)	Interindividual variability on absorption rate	155	(55.9–628)
*IIV Q* (*CV%*)	Interindividual variability on distributional clearance	25.7	(7.74–42.0)
*IIV N* (*CV%*)	Interindividual variability on number of transit compartments	20.2	(7.57–38.2)
*IOV MTT* (*CV%*)	Inter-occasional variability on mean transit time	4.39	(2.31–6.18)
*IOV N* (*CV%*)	Inter-occasional variability on number of transit compartments	17.6	(9.32–25.2)
**𝝈^2^−*Estimates***			
*PRV* (*CV%*)	Proportional residual variability	19.4	(16.0–22.9)

a CI, confidence interval (nonparametric) based on 2.5 and 97.5% percentiles obtained by the bootstrap analysis based on the final model applied to the original dataset; F, bioavailability.

*CV% for IIV and IOV computed as* $\sqrt{\exp\left(\omega^{2}\right)-1}$*; CV% for PRV computed as* $\sqrt{\exp\left(\sigma^{2}\right)-1}$.

### Effect of Baicalin Co-administration on the Pharmacokinetics of Cyclosporine A

**Figure 4:**
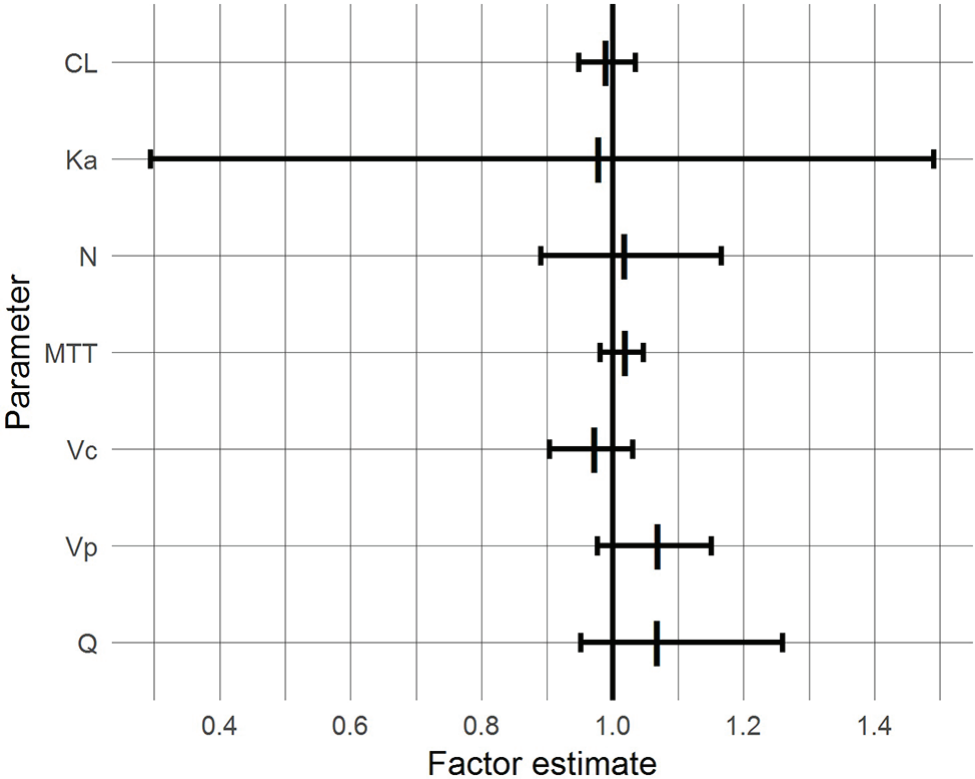
95% confidence intervals (bars) and medians (vertical lines) of BG effects based on a bootstrap with 1,000 samples.

With the exceptions of Ka and Q, the 95% CIs of the factor “BG co-treatment” on the parameters included 1.0 and were inside the 0.8–1.25 range, suggesting that BG did not affect the respective pharmacokinetic parameters of CsA to a clinically relevant extent (Figure 4). For Ka and Q, the CIs also included unity but were wide and exceeded the range, reflecting pronounced interindividual variability.

### Safety and Tolerability

No severe or serious adverse events were observed and all subjects were in good health. All participants completed the study with adequate compliance and no subject dropped out of the study.

Forty-three adverse events occurred in 13 subjects after administration of CsA (Table 4). In the first reference period, 11 subjects suffered 20 events, especially abdominal discomfort, which was the most frequently reported drug-related AE. In the second treatment period, 13 subjects reported 23 events where heartburn, accounting for 21.7% of events in this period, was most frequently reported. All but one AE in the two periods were considered as related to the study medication. No notable change was recorded in the vital signs or clinical laboratory variables when comparing baseline and end of study evaluations. Besides, there were no clinically relevant changes in ECG in individuals during the study. All the AEs reported were mild and resolved without dose interruption, treatment, or sequelae.

**Table 4:** Summary of adverse events in the clinic trial.

Adverse events	Number of events (CsA alone)	Number of events (CsA + BG)
Headache	–	1
Nausea	3	3
Dizziness	1	–
Altered taste	1	–
Abdominal discomfort	4	2
Heartburn	3	5
Mouth ulcer^a^	–	1
Feeling hot	3	4
Oesophagitis	1	1
Pharyngitis	3	3
Palpitation	1	–
Chest congestion	–	3

a Which was considered unlikely to be related to the study drug.

## Discussion

In this study, non-compartmental analysis of the blood concentration vs. time profiles of CsA indicated that co-administration of a single 500 mg BG dose has no clinically relevant effect on the exposure of CsA in healthy volunteers. The compartmental population pharmacokinetic analysis further confirmed this result for underlying pharmacokinetic processes.

After oral administration, BG, due to low lipophilicity, may either be directly absorbed by the action of uptake transporters, or undergo hydrolysis by intestinal glucuronidase or intestinal microflora to release its aglycone B (Akao et al., 2010; Kang et al., 2014; Noh et al., 2016; Kalapos-Kovács et al., 2018). B is probably better absorbed and then efficiently conjugated to BG in the gut wall and the liver and thus restored to its original form BG (baicalein 7-O-glucuronide) as well as to baicalein 6-O-glucuronide (Liu et al., 2009; Zhang et al., 2011). Thus, both BG and B would be present at the various locations of CYP3A4 and P-gp in the gut wall and the liver, with the potential to modify their activity and/or expression (Morisaki et al., 2013; Miao et al., 2016), if sufficient concentrations were reached.

However, an effect of BG on the pharmacokinetics of CsA was not observed in this study based on all but two of the assessments. The observation in the population pharmacokinetic analysis that 95% CIs for Ka and Q exceeded the “no relevant effect” range probably just reflects that the study was not powered to quantify an effect on these parameters. There are three potential explanations for the finding that BG did not cause a drug–drug interaction (DDI). First, formation of B, the much more active moiety to cause DDIs (Morisaki et al., 2013; Xin et al., 2013; Tian et al., 2019), from BG may be very limited in humans *in vivo*. Second, even if B would be generated extensively, it is subject to (re-)glucuronidation in the gut, the gut wall, and/or the liver (Akao et al., 2010), thus losing (most of the) effects on CYP3A4 and P-gp. Indeed, when B single doses were administered directly to healthy volunteers, exposure to BG exceeded that to B more than 10-fold (Li et al., 2014), indicating that glucuronidation of B in humans is rapid and extensive. Third, it cannot be excluded that competing mechanisms of inhibition would cancel each other out, but the different time courses of inhibition of drug metabolizing enzymes, transporter, or protein binding vs. induction by increased protein synthesis make this explanation unlikely. In summary, independent of the underlying mechanism, it appears that the systemic exposure of B produced after a single oral administration of BG in this study was too low to affect the activities of CYP3A and P-gp.

The present study is the first to study any DDI of BG in humans. One study in rats reported that single dose of BG (136.6 mg/kg) markedly elevated the *C*_max_ and AUC of CsA to about 4 times and 6 times, respectively, compared with CsA administered alone (Lai et al., 2004). However, our recent study in rats showed that multiple intravenous doses of BG did not affect the exposure of CsA but multiple oral administrations of BG (80 mg/kg) could decrease the *C*_max_ and AUC_0-∞_ of CsA by 38 and 25%, respectively (Tian et al., 2019). Further study resulted in that after multiple oral doses of BG treatment, the expression of P-gp of rats increased in the intestine, but was not changed in the liver (Tian et al., 2019). The different results of the two studies in rats might be related to the dosage, and the different results between rats and humans might be caused by the species differences, particularly in the formation and re-glucuronidation of B.

The additional use of population pharmacokinetic analysis might serve as a powerful tool to improve the understanding of potential DDI. First, the non-compartmental analysis might result in poorly estimated parameters such as clearance and volume(s) of distribution and confound sources of variability such as interindividual, intraindividual, and inter-occasion variability based on the actual observation instead of basic pharmacokinetic parameters as the dependent variable. Secondly, the population pharmacokinetic approach enables to assess PK processes underlying drug exposure separately. This way, it was possible to show that neither clearance nor volumes of distribution of CsA, which are the most important PK parameters to describe exposure, were affected by BG. As a limitation, it was not possible to separately describe intestinal and hepatic metabolism of CsA and thus to assess interaction at these two sites separately; to this end, both oral and intravenous administration of CsA would have been required (Gazzaz et al., 2018).

Pharmacokinetic data of BG in humans are sparse. Published data include the pharmacokinetics of BG after oral administration of BG to healthy subjects in a bioequivalence study (Wu et al., 2005), and pharmacokinetics of BG and B after single and multiple oral doses of B administered to healthy volunteers (Li et al., 2014; Pang et al., 2016). The AUC of BG after oral administration of 750 mg BG (613 ng h ml^−1^) (Wu et al., 2005) was similar to that after oral administration of only 100 mg B (580 ng h ml^−1^) (Li et al., 2014), which would be compatible with poor bioavailability of BG; unfortunately, in the study with BG administration, B concentrations were not quantified. Furthermore, BG was absorbed very slowly, with a *T*_max_ of 7.4 h (Wu et al., 2005), while B was absorbed much faster with a *T*_max_ of about 1 h for both BG and B (Li et al., 2014). These data suggest that both extent and temporal course of systemic concentrations of BG and probably its metabolite B in the present study were not sufficient to mediate an effect on CsA pharmacokinetics.

Although the current study would not suggest that dose adjustment might be warranted when BG and CsA are co-administered, because of the limited information on BG pharmacokinetics, a mechanistic extrapolation to other settings such as chronic BG dosing, higher BG doses, different timing of BG doses, or administration of B instead of BG would not be possible. Thus, in additional studies, BG and B plasma and/or blood concentrations should be quantified. Secondly, multiple dosages of BG should be used to treat the subjects before the administration of CsA to achieve maximal BG/B exposure as the single treatment of BG might not have been enough to modify CYP3A4 and/or P-gp. Furthermore, while in this study, the pharmacokinetic parameters of CsA were consistent with other healthy volunteer studies of CsA (Garg et al., 2011), clearance of CsA in healthy volunteers was two times higher than that of kidney or liver transplant patients (Bo et al., 2010), suggesting that DDI results from healthy volunteers might not be directly extrapolated to organ transplant patients. Thus, future investigations to evaluate the effect of chronic BG administration on CsA pharmacokinetics in patients would also be of interest. Finally, CYP3A5 genotype affects the clearance of CsA (Song et al., 2012). Thus, subjects with different CYP3A5 genotypes would be preferable to be recruited for subsequent studies.

Both the pharmacokinetic data and the limited safety data of the present study do not provide evidence that BG co-administration with CsA would cause an additional risk. The possibility of co-treatment with these drugs for transplant patients might be beneficial, because BG could exert antiviral, anti-inflammatory, anti-oxidation, and anti-proliferation effects (Lee et al., 2015; Ming et al., 2018). Indeed, in an observational study, it was reported that when BG was given with the antiviral drug telbivudine for the treatment of hepatitis in the clinic, the liver function (ALT) normalization rate, HBV DNA and hepatitis B virus markers (HBeAg) negative conversion rate, and anti-HBe serum conversion rate in the treatment group (BG and telbivudine, *n* = 64) were significantly higher compared to the reference group (telbivudine alone, *n* = 62) (Fang et al., 2011). Thus, these first results on a potential DDI between BG/B and CsA are encouraging and should be expanded by further studies as described above.

## Conclusion

In summary, this is the first clinical study to investigate the effect of BG on CsA pharmacokinetics in humans. The current dosage of BG (500 mg single dose) and CsA (200 mg single dose) was generally safe and well tolerated in the adult subjects without serious adverse events observed. Both non-compartmental analysis and the population pharmacokinetic approach did not provide any evidence that the exposure to CsA and/or underlying pharmacokinetic processes of CsA were changed to a clinically relevant extent by BG. These results need to be confirmed in studies with maximal chronic exposure to BG and quantification of BG and B.

## Ethics Statement

This study was carried out in accordance with the standards of Good Clinical Practice, all applicable regulations, specific legal requirements, and ethical principles as described in the Declaration of Helsinki with written informed consent from all subjects. All subjects gave written informed consent in accordance with the Declaration of Helsinki. The protocol was approved by the Ethics Committee of at the first affiliated hospital of Zhengzhou University (Henan, China; approval no. SR201509).

## Author Contributions

XT and XZ designed and supervised the clinical trial. RL, JW, SL, YC, JiZ, and JiaZ carried out the clinical trial. RL, JW, and XL performed the non-compartmental analysis. XL conducted the population pharmacokinetic analysis. XL, RL, UF, and JW wrote the manuscript. UF, MT, and XT edited the manuscript.

### Conflict of Interest Statement

The authors declare that the research was conducted in the absence of any commercial or financial relationships that could be construed as a potential conflict of interest.
